# Senescence-Associated miRNAs and Their Role in Pancreatic Cancer

**DOI:** 10.3389/pore.2022.1610156

**Published:** 2022-04-29

**Authors:** Alexey Popov, Vaclav Mandys

**Affiliations:** Department of Pathology, Third Faculty of Medicine, Charles University and University Hospital Kralovske Vinohrady, Prague, Czechia

**Keywords:** oncogene, pancreatic ductal adenocarcinoma, cellular senescence, senescence-associated miRNA, senescence bypass, tumor suppressor

## Abstract

Replicative senescence is irreversible cell proliferation arrest for somatic cells which can be circumvented in cancers. Cellular senescence is a process, which may play two opposite roles. On the one hand, this is a natural protection of somatic cells against unlimited proliferation and malignant transformation. On the other hand, cellular secretion caused by senescence can stimulate inflammation and proliferation of adjacent cells that may promote malignancy. The main genes controlling the senescence pathways are also well known as tumor suppressors. Almost 140 genes regulate both cellular senescence and cancer pathways. About two thirds of these genes (64%) are regulated by microRNAs. Senescence-associated miRNAs can stimulate cancer progression or act as tumor suppressors. Here we review the role playing by senescence-associated miRNAs in development, diagnostics and treatment of pancreatic cancer.

## Introduction

Replicative senescence is irreversible cell proliferation arrest. Senescent cells stop their divisions, grow in size and start specific secretory activity. This process often results from somatic cells aging and telomeres shortening. The same state may be provoked by DNA damage, oncogenesis etc. Activity of oncogenes and pro-proliferative genes may promote expression of *TP53* gene, well known as a tumor suppressor, and induce cellular senescence and/or apoptosis. Most senescent cells also express another tumor suppressor gene, *p16INK4a*. Thus, oncogene-induced senescence is a natural barrier for tumorigenesis. On the other side, senescent cells produce growth factors, proteases and cytokines which are necessary for the tissue renewal. Deregulated secretion of these factors can provoke malignant transformation after different premalignant damages and in benign tumors. There is a group of genes, which are necessary for both cellular senescence and carcinogenesis. The majority of these genes are regulated by microRNAs. These miRNAs regulating cellular senescence may act as tumor suppressors or stimulators. This review is focused on the role playing by senescence-associated miRNAs (SA-miRs) in development of pancreatic cancer, which is one of the most aggressive oncogenic diseases.

## Cellular Senescence and Cancer

Almost 60 years ago, Hayflick described cellular senescence as a process blocking replicative potential and growth of human diploid fibroblasts in culture. As was found, human fibroblasts change their morphology and stop to divide after 50–60 rounds of cell divisions. This phenomenon is known as replicative senescence, or the Hayflick limit [1]. In contrast with the normal somatic cells, cancer and embryonic stem cells can escape the cellular senescence [2–4]. Senescent cells undergo some morphological changes, for example, they increase in size, more than twofold and form heterochromatin foci inside their nucleus. Besides, these cells start specific secretory activity (senescence-associated secretory phenotype, SASP) [5, 6].

Senescence can be also a cellular response to different damaging agents, chemical or physical. Many factors may trigger cellular senescence. The telomere shortening during replication is particularly important. Extremely short telomeres as well as DNA damages result in DNA damage response (DDR), a chain of events starting cellular senescence in G1 phase [6–9]. The molecular basis of this G1 arrest is thought to be due to a DNA damage response, resulting in accumulation of the cyclin dependent kinase (CDK) inhibitors p21 and p16 that block the inactivating phosphorylation of the retinoblastoma tumor suppressor pRb, thereby preventing DNA replication. Protein p21 acts downstream of p53 whereas p16 acts upstream of pRB. As was shown, p21 also mediates permanent DNA damage-induced cell cycle arrest in G2 (G2 exit) by inhibiting mitotic CDK complexes and pRb inactivation [10, 11].

Loss of tumor suppressors (*ARF, TP53*, and *PTEN*) or active expression of oncogenes (*KRAS, BRAF* and *MYC*) in normal cells also promotes cellular senescence. This phenomenon is known as oncogene induced senescence (OIS) [5, 6, 9]. It was first observed when an oncogenic form of *RAS*, a cytoplasmic transducer of mitogenic signals, was expressed in normal human fibroblasts [12].

Both DDR and OIS activate one of the main pathways for cellular senescence, *INK4a/ARF* cascade [9, 13]. *INK4a* locus expresses two small proteins: p16INK4a and p19ARF (alternative reading frame). Cyclin-dependent kinase inhibitor (CDKI) p16INK4a prevents pRB (retinoblastoma protein) phosphorylation and inactivation, which leads to cellular senescence. Another protein, p19ARF, cooperates with p53 bringing about cell cycle arrest and subsequent senescence [9, 13, 14]. All genes of *ARF* cascade are well known tumor suppressors blocking cell cycle progression during malignant transformation. Products of these genes produce a barrier that prevents carcinogenesis. Accordingly, these genes are often inactivated by mutations or promoter methylation in different tumors, such as breast, colon, liver and pancreatic cancer [15–18].

There are several pathways triggering or regulating cellular senescence, but their deregulation results in tumor development. The phosphatidylinositol 3-kinase (PI3K)/AKT pathway constitutes an additional route to the establishment of OIS since it promotes mTOR-regulated translation and stabilization of p53 [19, 20]. Loss of tumor suppressor *PTEN*, negative regulator of PI3K/Akt pathway, may promote cancer progression. It is estimated that in at least 50% of all cancer patients PI3K/Akt signaling pathway is deregulated [20]. Another pathway, which involves transforming growth factor beta (TGF-β), blocks cell cycle progress through G1 phase. TGF-β causes senescence stimulating synthesis of p15 and p21 proteins and prevents Rb phosphorylation. On the other hand, constant TGF-β expression is necessary for cancer cell migration and invasion [6, 21, 22]. Nuclear factor kappa light-chain-enhancer of activated B cells (NF-κB) participates in a senescence-associated cytokine response and control of SASP components secretion which suggests a tumor restraining role of NF-κB. On the other hand, constitutive aberrant activation of NF-κB has been observed in different kinds of cancer, including lymphoma, leukemia, breast, colon, liver, pancreas, prostate, and ovarian cancers [4, 23]. Notch signaling pathway is involved in cell-contact-dependent juxtacrine senescence, where cells are characterized by distinct SASP components [24, 25]. In cancers aberrant *NOTCH* activation correlates with activation of NF-κB and PI3K/Akt pathways which enhances tumor growth and resistance chemotherapy [26].

NF-κB [23, 27], mTOR [6, 28], and Notch [24, 29] pathways are involved in SASP regulation. Senescent cells secrete up to 80 specific substances including collagen and fibronectin, interleukins (in particular IL-1, IL-6 and IL-8), growth factors and metalloproteases. These factors are necessary for tissue renewal [6, 30, 31]. Cellular senescence can be transmitted to neighboring cells through secreted SASP factors (including IL-1 and Notch ligands) thus it prevents the malignant transformation. IL-6 and IL-8 promote inflammation leading to the recruitment of lymphocytes and macrophages to eliminate senescent and premalignant cells [32–34]. On the other hand, deregulated persistent SASP factors secretion produces a chronic inflammatory microenvironment in tissues and can induce malignant transformation in neighboring cells. Pro-inflammatory cytokines IL-6 and IL-8 can stimulate epithelial-mesenchymal transition (EMT), cell migration and invasion [8, 34]. SASP turns senescent fibroblasts into pro-inflammatory cells with the ability to promote EMT and tumor progression [35]. Additionally, senescent fibroblasts and mesothelial cells secrete vascular endothelial growth factor (VEGF) inducing neovascularization as well as matrix metalloproteinases which facilitate tumor cell migration and invasion [8, 36, 37]. Thus, SASP acts in a context dependent manner and has either pro- or anti- tumorigenic effect.

Therefore, cellular senescence is a process, which may act in two opposite directions. On the one hand, senescence is a natural mechanism of somatic cells protection against unlimited proliferation and malignant transformation. The main genes, controlling the senescence pathways, are also well known as the tumor suppressors. Their loss or aberrant expression helps malignant cells to bypass senescence and promotes cancer progression. Besides, senescent cells produce secretory factors, which are necessary for cancer cells elimination and the tissue renewal. On the other hand, aberrant SASP secretion can stimulate inflammation and carcinogenesis.

## Cellular Senescence in Pancreatic Cancer

Senescent pancreatic cells have first been detected in low grade pancreatic intraepithelial neoplasias (PanINs) in the mouse models expressing oncogene *KRAS* from its endogenous promoter [38]. More than 90% of pancreatic ductal adenocarcinomas (PDACs) harbor *KRAS* activating mutations [39, 40]. Active *KRAS* in the pancreas leads to development of premalignant lesions which display low proliferative activity and contain cells expressing markers of cellular senescence [41, 42]. Caldwell et al. found that about 10% of cells in mouse PanIN-1 are senescent and express the standard senescence marker SA-β-gal (senescence-associated β-galactosidase). These cells were negative for proliferative marker Ki67. The number of senescent cells in mouse PanINs was decreasing during the PanIN progression from grade 1 to 3. Senescent cells were also detected in human PanINs and PDACs but the number of these cells was much less than in the mouse models [41, 43]. High-grade mouse PanIN2/3 lesions as well as PDAC were negative for senescence markers including endogenous senescence-associated β-galactosidase and expression of the p16INK4a [41, 43]. Moreover, another cell subpopulation (about 10%) expressing both Ki67 and SA-β-gal was detected in murine PanINs [41]. Deschênes‐Simard et al. found that mouse PDAC-derived cell lines exhibit stem cells properties, while PanIN‐derived cell lines do not. These findings indicate that cancer cells can escape senescence and reentry in the cell cycle and proliferation through the reprogramming from senescent to “stem” cell status [42].

Senescence may also be bypassed by a number of mutations inactivating most important genes of senescence pathways. Tumor suppressors *TP53* and *CDKN2A/INK4* harbor mutations in 80% and 85% of PDACs correspondently [18]. Deletion of *Rb* accelerates pancreatic carcinogenesis driven by oncogenic *KRAS* expression and impairs senescence in premalignant lesions [44]. *SMAD4*, a member of TGF-β pathway, is deleted in 50% PDACs [18]. In almost 60% of all PDAC patients the PI3K/Akt signaling pathway is deregulated [20, 45]. Loss of *PTEN* (PI3K inhibitor) expression in 25–70% of PDAC cases correlate with the short-term overall survival [20].

Some of tumor suppressors can also be inactivated by epigenetic alterations. Altered gene methylation, regulated by DNA methyltransferases (DNMT) 1, 3a and 3b, contributes to PDAC development [46]. *DNMT1*, *3a* and *3b* were expressed in 46.6%, 23.9%, and 77.3% of PDAC tissues, respectively, but not in normal pancreas [47]. *CDKN2A* (*INK4a*) locus may be inactivated by hypermethylation in 18% of PDACs [48]. Overexpression of *DNMT1* was believed to be responsible for silencing key tumor suppressor genes including p16 [49]. Histone deacetylase SIRT1 has been shown to be involved in the deacetylation of non-histone proteins such as p53, Rb, and Smad7, allowing cells to bypass senescence and survive DNA damage [3].

Analysis of all exons and selected introns of 410 cancer-associated genes was performed in tumor samples from 336 PDAC patients demonstrated frequent gene alterations of several pathways, including TGF-β, Notch and NF-κB signaling, which are associated with cellular senescence and SASP regulation but can stimulate cancer aggressiveness, chemoresistance and metastasis in PDACs [37, 50]. NF-κB, a major transcription factor involved in these inflammatory responses, is found to be activated in KRAS-transformed epithelial cells. In mouse models it also has been shown that interaction between NF-κB and Notch signaling pathways is needed to drive a sustained inflammatory response [51, 52].

Certain pathological stimuli, such as inflammation, also seem appear to promote tumorigenesis in PDAC by means of a senescence bypass [4, 43]. Senescent cells secrete interleukins (in particular IL-1 and IL-6), growth factors and metalloproteases that stimulate inflammation leading to the recruitment of lymphocytes and macrophages for elimination of premalignant cells [3, 4, 53]. On the other hand, persistent or deregulated SASP activation can promote chronic inflammation and therefore drive cancer progression [4, 54]. In chronic pancreatitis, the number of senescent cells significantly correlates with the severity of inflammation and fibrosis. Both the fibrotic region and senescence-associated SA-β-gal positive region overlap with the region densely infiltrated by immune cells [55]. Senescent cells are also accumulated in tumor microenvironment, including carcinoma-associated fibroblasts and activated pancreatic stellate cells [55–57]. Both these cell subpopulations produce SASP factors which may contribute to cancer development and metastasis [57, 58]. The role that senescent cells play in formation of the inflammatory PDAC microenvironment remains for the most part unknown [3, 4, 56].

Therefore, in chronic pancreatitis or PanIN of low grade cellular senescence may prevent malignant transformation. Under conditions of chronic inflammation pancreatic cells may accumulate mutations inactivating key senescence pathways and thus start tumor development. However, the mechanism of senescence bypass in tumors that spontaneously arise from premalignant lesions remains mostly unclear. SASP possibly may play a dual role in pancreatic carcinogenesis: at the beginning it recruits immune cells for elimination of the malignant cells, but later it provokes persistent inflammation and supports tumor progression.

## SA-miRs in Pancreatic Cancer

According to the data Tacutu et al., more than 262 human genes are associated with cellular senescence. More than a half of the senescence-associated genes (138 genes) participate in both cellular senescence and cancer pathways [59]. Almost two thirds of these genes (64%) are regulated by microRNAs. MicroRNAs (miRNAs) are a class of single-stranded RNA molecules of 15–27 nucleotides in length that regulate gene expression at the post-transcriptional level. Initially, miRNAs are transcribed as thousand-base-long primary transcripts by RNA polymerase II and are called precursor miRNAs. Precursor miRNAs are exported to the cytoplasm via exportin 5, where they are integrated into DICER and RNA-induced silencing complex (RISC). MicroRNAs use the RISC complex on their mRNA targets for translational repression or degradation [60].

Tacutu et al. detected approximately 40 miRNAs regulating expression of both senescence-associated and cancer-related genes [59]. The senescence-associated miRNAs (SA-miRs) control cell transition during cell cycle, mainly through the G1/S or G2/M checkpoints by targeting cyclin-dependent kinases (CDKs) and cyclin-dependent kinase inhibitors (CDKIs) [61].

More than 25 senescence-associated miRs (SA-miRs) were identified in pancreatic cancer. Pancreatic tumors demonstrate very low number of senescent cells, but PDAC cells produce SA-miRs stimulating processes of carcinogenesis, tumor growth and survival as well as cancer microenvironment formation [59]. These miRNAs are often packed into exosomes which can deliver functional SA-miRs to recipient cells. Exosomes are membrane-bound extracellular vesicles (EV) containing biological materials (proteins and nucleic acids) and play an important role in communication among cells. This kind of EVs originates by the release of intraluminal vesicles (ILVs) after fusion of multivesicular bodies (MVBs) with plasma membrane. MVBs move toward the plasma membrane to fuse and release ILVs that, in extracellular space, become exosomes. Target cells uptake these miRNAs by endocytosis or pinocytosis then release them from microvesicles [62, 63]. Exosomal miR-155 and miR-210 can increase PDAC resistance to chemotherapy. SA-miRs of miR-200 family stimulate cancer cells migration and invasion. Highly elevated levels of miR-17-5p and miR-21 stimulating cancer cells proliferation were detected in serum samples of pancreatic cancer patients [62, 64].

Cancer cells release extra-cellular miRNAs to recruit macrophages for the tumor microenvironment formation. One more function of these EVs is to “educate” the immune system to spare PDAC cells from active killing [64]. Moreover, exosomes released by cancer cells can travel to distant organs, such as the liver and brain, and can modulate the microenvironment to establish a metastatic niche and subsequent metastasis [65].

EVs are implicated in the transformation of various precancerous lesions into PDAC and in the progression of cancer toward more invasive and metastasizing forms. Inside these lesions cells produce exosomes containing miR-21, miR-155 and 210 which promote inflammation as well as pancreatic stellate cells activation [62, 66]. Vicentini et al. located by *in situ* hybridization that exosomal SA-miRNAs including miR17-5p were derived from the epithelial components of the lesions [67].

In contrast with PDAC, anti-oncogenic SA-miRs are constantly expressed in normal pancreatic tissues. These miRNAs, such as miR-146a and miR-217, demonstrate high expression levels not only in the senescent cells [68, 69]. As a component of SASP, exosomes of the senescent cells can include two opposite sets of SA-miRs, both senescence-inducing (let-7a, miR-34 and miR-217) and pro-oncogenic (miR-21, miR-155 and miR-221) [70, 71].

Accumulating evidences showed that pancreatic tumor cells communicate with stromal cells in the local environment or even in the remote organs *via* secretion of extracellular vesicles packed with SA-miRs. Stromal cells that lack genomic instabilities uptake these miRNAs then release from microvesicles into the target cells as messengers to dictate them so as to facilitate tumor progression and metastasis [72, 73]. Pancreatic cancer-secreted SA-miRs, such as miR-21, miR-155 or miR-210 implicates in the conversion from normal fibroblasts to cancer-associated fibroblasts (CAF) [74, 75]. Also, exosomes containing SA-miRs can promote EMT as well as convert pancreatic stellate cells and bone marrow-derived stem cells into the CAF [76]. In turn, CAF release a variety of circulating SA-microRNAs including miR-21, miR-210 etc. which stimulate cancer cells proliferation, migration and invasion as well as support angiogenesis, and recruit monocytes/macrophages [74]. Senescent CAFs, like other senescent cells, present a SASP composed of pro-tumorigenic factors. Senescent cells produce exosomal miR-21, miR-146, miR-155a, miR-210 and miR-221 stimulating inflammation process aa well as cancer cells proliferation, migration and invasion [77]. In addition, the existence of a senescent CAF population in PDAC endowed with invasion- and metastasis-promoting properties as well as poor patient prognosis [78].

SA-miRs often display aberrant expression levels in tumors. Abnormal expression of miRNAs is one of important clinical markers for PDACs diagnostics and treatment. A list of SA-miRs [37, 59, 79], deregulated in pancreatic cancers, are presented in Tables 1, 2.

**TABLE 1:** Senescence-associated oncomirs in pancreatic tumors.

Oncomirs (upregulated)	miRNA targets	SA-miR enhances	References
miR-10b	*TIP30*	Invasion	[80, 89]
miR-15b	*SMURF2*	Metastasis	[90]
mir-17-5p	*RBL2* and up to 20 cell cycle regulators	Proliferation, invasion	[91–94]
miR-21	*CDK2AP1*, *Pdcd*, *BCL2*, *PTEN* and almost 30 genes	Proliferation, invasion, chemoresistance, tumor survival	[81–83]
miR-155	*TP53INP1*, *FOXO3a*, and *SOCS1*	Proliferation, transformation, migration and invasion	[95–97]
miR-181a	*PTEN*	Proliferation	[84]
miR-210	*Ephrin-A3, MNT*	Proliferation, angiogenesis, tumor growth and survival	[98–101]
miR-221	*TIMP2*, *PTEN*, *p27*(*kip1*), *p57*(*kip2*)*, and PUMA*	Proliferation, invasion	[82, 83]
miR-222	*TIMP2*	Proliferation, invasion	[82]

**TABLE 2:** Tumor-suppressing SA-miRs in pancreatic cancer.

Tumor suppressors (downregulated)	miRNA targets	SA-miR inhibits	References
let-7 family	*KRAS*, *HMGA2*, *CDC25a*, *CDC34*, *CDK6*, *BCL2*	Proliferation, ^a^EMT, metastasis, chemoresistance	[104–106]
miR-24-3p	*LAMB*	Migration, invasion	[115]
mir-26b	*CDK14*	Proliferation	[116]
miR-29a	*MUC1* and *LOXL2*	Proliferation, migration, chemoresistance	[117–119]
miR-30a	*SNAI1*	Proliferation, tumor survival, chemoresistance	[120]
miR-34a	*NOTCH*, *BCL2*, *VEGFA*, *CCND1*, and *CDK6*	Proliferation, angiogenesis, EMT, metastasis	[107–112]
miR-107	*CDK6, PI3K/AKT*	Proliferation, metastasis	[113, 114]
mir-124	*IL6R*, *STAT3*, *MCT1*	Proliferation, tumor growth	[110, 111, 124]
miR-126	*ADAM9*	Invasion, EMT	[133]
miR-137	*KDM4A*	Proliferation	[122, 123]
miR-141	*MAP4K4*	EMT, metastasis	[125–127]
miR-145	*MUC13*, *NEDD9*	Invasion, EMT	[134]
miR-146a	*IRAK-1*	Migration, invasion	[135, 170]
miR-148a	*PHLDA2*, *LPCAT2*, and *AP1S3*	Proliferation, migration, invasion	[122]
miR-200 family	*ZEB1, ZEB2*	EMT, migration, invasion	[69, 128, 136]
miR-217	*KRAS, SIRT1*	Proliferation	[132]
miR-335	*OCT4*	Proliferation	[129]
miR-494	*SDC1*	EMT, metastasis	

a EMT, epithelial-mesenchymal transitions.

SA-miRs playing an important role in pancreatic tumors formation and development can be classified into two major groups: oncomirs and cancer suppressors. The first group of SA-miRs stimulates proliferation and migration of cells, chemotherapy resistance and metastasis (Table 1). The second group of miRNAs activates genes of cellular senescence and apoptosis pathways; thereby functioning as tumor suppressors (Table 2).

## Oncogenic SA-miRs Promote Pancreatic Cancer

A large number of SA-miRNAs are overexpressed in pancreatic cancer. Nakata et al and Eun et al. reported that miR-10b, miR-155, miR-21, miR-221 and miR-222, were aberrantly expressed in PDAC [80, 81]. MiR-21 is one of the first identified cancer-promoting oncomirs, which targets almost 30 genes, including tumor suppressors, such as *CDK2AP1*, *Pdcd4* and *BCL2* [82]. *PTEN*, which suppresses PI3K-AKT-mTOR senescence pathway, is also a target for miR-21 as well as miR-181a and miR-221 [83, 84]. High expression levels of miR-21 were detected in early pancreatic ductal adenocarcinoma precursor lesions [85]. MiR-21 stimulates PDAC cell proliferation, invasion, chemoresistance and prevents apoptosis [83, 85–88]. MiRNA-10b enhances pancreatic cancer cell invasiveness by suppressing *TIP30* expression and promoting EGF and TGF-β effects [80, 89]. MiR-15b degrades *SMURF2* transcripts, which is also participant of TGF-β pathway, and this miRNA expression was associated with enhanced metastasis in PDACs [90]. MiRNA-17-5p negatively regulates more than 20 genes involved in the G1/S-phase transition [91, 92]. Overexpression of this miRNA in pancreatic cancer is associated with intensive cancer cell proliferation and invasion as well as poor prognosis [93, 94]. MiR-155 is inhibitor of tumor protein 53-induced nuclear protein 1 (*TP53INP1*) and *FOXO3a* expression, leading to cell proliferation and malignant transformation [95, 96]. Also miR-155 is associated with the JAK/STAT pathway, it negatively regulates *SOCS1* and accelerates migration and invasion of PDAC cells [97]. Mir-210 is necessary for tumor angiogenesis, cell cycle regulation and cancer survival in hypoxia conditions [98–101]. MiR-221 and 222 genes are placed in tandem on the X chromosome. Activity of these miRNAs stimulates cancer cells proliferation and invasion [102, 103].

The cited works show that SA-miRNAs may control expression of several groups of tumor-suppressor genes from various pathways. Most of them act like the inhibitors of main senescence or apoptosis pathways, such as p53-p16-pRB or PTEN-PI3K-AKT-mTOR. Thus, SA-oncomirs are necessary for successful PDAC cell proliferation, chemoresistance, survival and tumor progression.

## SA-miRNAs May Act as Tumor Suppressors in Pancreatic Ductal Adenocarcinomas

Another group of the SA-miRs are often downregulated in PDACs by DNA methylation or gene loss. These miRNAs may inhibit cell proliferation; prevent cancer cells chemoresistance, migration and invasions besides they induce cellular senescence and apoptosis. For example, miRNA family let-7 inhibits cancer cell proliferation, metastasis and chemoresistance [104–106]. MicroRNA-34a is a tumor suppressor, like let-7, and a promising candidate for pancreatic cancer therapy [107]. There are multiple target genes for miR-34a, such as *NOTCH*, *BCL2*, *VEGFA*, *CCND1* and *CDK6*, regulating cell cycle, p53/p38-MAPK, Notch and PI3K/Akt pathways [108–112]. Tumor suppressing miR-107 also inhibits *CDK6* and stimulates *PTEN* expression [113, 114]. MiR-24-3p downregulates laminin subunit beta 3 (*LAMB*), inhibits processes of cancer cells attachment and migration, modifies their interaction with other extracellular matrix components [115]. MiR-26b directly inactivates cyclin-dependent kinase *CDK14* in cancers. Expression of *CDK14* promotes cancer cell aggressiveness [116]. The data about miR-29a are controversial. MiR-29a, targeting M*UC1* and *LOXL2*, inhibits cell proliferation, migration, invasion and sensitize pancreatic cancer cells to gemcitabine [117, 118]. On the other hand, miR-29a may stimulate pancreatic cancer growth by inhibiting the expression of tristetraprolin [119]. MiR-30a regulates cancer cell response to chemotherapy through SNAI1/IRS1/Akt pathway, which is fundamental in mediating multiple processes, including cell proliferation and survival, angiogenesis and glucose metabolism [120]. There is a group of SA-miRs, involved in pancreatic cancer stem cells regulation, inhibition of epithelial-mesenchymal transitions (EMT) as well as prevention of cancer cells migration and invasion [121]. This group includes two miRNA families: let-7 [104, 105] and miR-200 (including miR-141) [122, 123] as well as miR-34a [111, 121] miR-126 [124], miR-145 [125–127], mir-217 [128] and miR-494 [129]. Overexpression of miR-124 downregulates IL6-JAK2-STAT3 pathway and inhibits PDAC cells proliferation [130]. Mir-124 also may suppress PDAC growth by regulation of cancer lactate metabolism [131]. MiR-137 and miR-335 triggers p53, p16 and *KRAS*-induced cellular senescence in PDACs [132, 133] MiR-146a inhibits the invasive capacity of pancreatic cancer cells with concomitant downregulation of EGFR and the NF-κB regulatory kinase, interleukin 1 receptor–associated kinase 1 (IRAK-1) [134]. MiR-148a targets may affect cell cycle and apoptosis [135]. MiR-217 is significantly downregulated in PDAC tissues and cell lines. Dual-luciferase reporter assay revealed that *KRAS* mRNA is the direct target of miR-217. Overexpression of miR-217 in a PDAC cell line decreases *KRAS* mRNA levels, and inhibits cell proliferation [136]. On the other hand, miR-217 is usually expressed in normal pancreas [69], and can induce cellular senescence in fibroblasts [137].

Thus, there are two groups of SA-miRs with opposite functions: the first one promotes cells proliferation, tumor growth and metastasis, the second one stimulates cellular senescence and apoptosis in PDACs. In PDACs the oncomirs are overexpressed but the tumor-suppressing SA-miRs are downregulated.

## SA-miRs and Pancreatic Cancer Diagnostics and Patient Prognosis

For the last 20 years aberrant expression was detected in a great number of SA-miRs. Differential expression of SA-miR profiles has been well described in PDAC, with miRNAs isolated from various patient-derived specimens, including the peripheral blood, pancreatic tissue, and digestive juices [138, 139]. Oncomirs, such as miR-21, may be upregulated up to 6888-fold in PDACs in comparison with normal tissue. About up to 52-fold increase for miR-155 was described in PDACs [140]. On the other hand, tumor-suppressing miR-217, was downregulated up to 62.5-fold in diagnostic needle aspirates from surgical pancreatic cancer specimens [141]. Lee et al. have selected a set of four miRNAs including miR-10b, miR-210, miR-202-3p and miR-375, and these miRNAs differentiated mucinous cystic lesions from intraductal papillary mucinous neoplasms and PDAC with sensitivity of 100% and specificity of 100% [142]. Diagnostic kit detecting aberrant expression of miRNAs was developed to discriminate malignant tissues from pancreatic lesions. This kit, miRInform Pancreas (Asuragen, Inc. Austin, TX), uses miR-217 and miR-196a to differentiate PDAC from other benign conditions with sensitivity and specificity of 95% [143]. The clinical trials of this kit have not been completed yet.

Circulating SA-miRs are attractive objects of study because of their abundance, stability, and easiness of isolation and amplification with inexpensive and non-invasive techniques [138, 139]. These miRNAs expression levels are also deregulated in the blood samples of PDAC patients’. Vila-Navarro et al. described significant overexpression of let-7, miR-21, miR-155, miR-181a and miR-210 in PDAC patients plasma samples [144]. Wei et al. analyzed 27 published studies involving more than 2000 PDAC patients and found that miR-10b, miR-21, miR34a, miR-221 and miR-155 were often upregulated in serum- or plasma samples. Among them, miR-21 was the most frequently identified dysregulated miRNA [145]. Meta-analysis of 46 studies involving 4326 pancreatic cancer patients demonstrated that utilization of circulating SA-miRs such as miR-10b, miR-181a and let-7a distinguished PDAC patients from non-PDAC controls with sensitivity of more than 90%. The serum levels of miR-200a identify patients with PDAC from healthy controls with a sensitivity and specificity of >80% [146]. A significant difference between PDAC and healthy groups was observed for the expression of miR-21 and miR-34a in serum samples [147]. Serum miR-124 levels were significantly decreased in patients with PDAC. Serum levels of miR-124 distinguished PDAC from chronic pancreatitis and healthy control subjects [148].

SA-miRs, whether circulating or isolated from tissue samples may serve as predictors of PDAC patient outcome. High expression levels of SA-miRs, including miR-21 [88, 149, 150], miR-155 [151, 152] and also miR-210 [153, 154] may be used as predictors for the cancer chemoresistance as well as poor prognosis. Greither et al. have proposed a prognostic panel consisting of miR-155, -203, -210, and -222, where their elevated expression is a predictor of poor outcome [154]. Low serum levels of miR-124 were significantly associated with lymph node metastasis, tumor node metastasis (TNM) stage and shorter survival time after surgery [148]. Yu et al. analyzed plasma levels of miR-210 with RT-qPCR in a cohort of 31 PDAC patients. High miR-210 expression was significantly associated with improved survival [153].

On the other hand, there is still no clinically approved miRNAs-based PDAC diagnostic system. The possible reasons for this may be a great variability of PDAC cells as well as the gene variability within the human population. Expression levels of miRNAs may vary greatly (sometimes showing opposite results) among patients even in the same hospital as well as in the population of different regions or countries.

## SA-miRs as Agents for Pancreatic Cancer Therapy

Therapy of PDAC by SA-miRs is based on assumption that oncomirs should be inhibited whereas tumor suppressors need to be restored to proper levels. As a result, cancer cells should enter the state of cellular senescence, stop proliferation and metastasis. Artificial SA-miRs (so called mimics) are two-stranded hairpin molecules imitating tumor-suppressor miRNAs [68, 155], whereas anti-miRs are chemically modified antisense strands [oligonucleotides with 2′-sugar modifications or locked nucleic acids (LNAs)], designed for elimination of oncomirs in cancer cells [156, 157]. Another possible way to eliminate oncomirs is miRNA sponge. This “sponge” is a small vector expressing miRNA target sequence “soaking” oncomirs and preventing them from association with their targets. These vectors may carry binding sequences for several different miRNAs. Expression levels of this vector need to be carefully calibrated for effective miRNAs elimination [158, 159]. A serious problem in the miRNAs-based cancer therapy is a proper miRNAs selection. Because of cancer cells heterogeneity in PDAC, single miRNA may not suffice for the tumor elimination [135]. Obviously, it is necessary to select a group of several miRNAs. On the other hand, each member of this group may control up to 30 target genes which may increase the probability of side-effects. Therefore, a lot of bioinformatics analyses are needed to predict and specify the whole network of selected miRNAs targets.

Efficient delivery of miRNA for therapeutic purposes is also highly problematic. Low cellular uptake of RNA, degradation in the bloodstream, and rapid renal clearance are significant obstacles on the way to the successful delivery of miRNA [160]. There are three methods for miRNAs delivery into tumors. The first one is based on lipid nanoparticles. Liposomes are spherical lipid bilayers that mimic biological membranes. Cationic liposome is positively charged and the negatively charged DNA binds to it by electrostatic interaction. Cells uptake lipid nanoparticles by endocytosis [161, 162]. The second approach make use of different viruses as the delivery agents [163]. The third method uses cationic polymers or dendrimers. Cationic polymers such as poly-L-lysine (PLL), polyethyleneimine (PEI), and oligopeptides can form polyplexes with miRNAs by means of electrostatic interactions. They can exist as linear or branched polymers of varying length. Dendrimers are a type of highly branched synthetic polymers with a spherical shape [160]. All of these methods have a lack of tissue and tumor-specific selectivity. SA-miRs delivery into normal tissues may have destructive consequences. Perhaps using of tissue specific and tumor specific (telomerase) promoters will help to solve this problem [164, 165]. Another possible way is to bind lipid particles or polymers with different ligands for tumor-specific receptors [160].

The first-ever miRNA therapeutic drug called miravirsen for the treatment of hepatitis C virus (HCV) infection is in phase II of clinical trials. Miravirsen is a short locked nucleic acid (LNA) antisense sequence for miR-122 [166]. MiR-34a mimicking drug MRX34 based on a lipid nanoparticle delivery system was used in a Phase I clinical trial to treat solid tumors and hematologic malignancies. This study was terminated because of the drug’s side effects [155, 167]. The study of two SA-miRs-based systems for PDAC therapy was started as preclinical trials. The first system is based on using lipid particles and mir-34a and miR-143/miR-145 cluster carrying nanovector [161, 168]. The second one employs miR-34a nanovector with special delivery nanocomplexes [169]. Nevertheless, there has not been developed any clinically approved miRNAs delivery system yet.

Thus, a lot of obstacles should be overcome to use SA-miRs for both PDAC diagnostics and miRNAs-based therapy.

## Conclusion

The SA-miRs may play two opposing roles in PDAC formation: some of these miRNAs block cellular senescence pathways and promote pancreatic cancer, whereas other acts like tumor-suppressors inducing senescence and apoptosis. Both these groups demonstrate abnormal expression levels which may be useful for PDAC diagnostics and patients prognosis. SA-miRs seem to have a great therapeutic potential as an instrument of decreasing chemoresistance of PDACs and preventing cancer cells proliferation, migration and invasion. But for the present there has not been established any clinically approved SA-miRs-based systems for diagnostics or therapy. Thus, future investigations are needed to resolve these problems.

## Abbreviations

EMT, epithelial-mesenchymal transitions; PanIN, pancreatic intraepithelial neoplasia; PDAC, pancreatic ductal adenocarcinoma; SA-miRs, senescence-associated microRNAs; SASP, senescence-associated secretory phenotype.
